# Imaging the Magnetization of Single Magnetite Nanoparticle Clusters via Photothermal Circular Dichroism

**DOI:** 10.1021/acs.nanolett.2c00178

**Published:** 2022-04-14

**Authors:** Patrick Spaeth, Subhasis Adhikari, Kaveh Lahabi, Martin Dieter Baaske, Yonghui Wang, Michel Orrit

**Affiliations:** † Huygens-Kamerlingh Onnes Laboratory, Leiden University, 2300 RA Leiden, The Netherlands; ‡ School of Mechatronics Engineering, Harbin Institute of Technology, Harbin 150001, P. R. China

**Keywords:** photothermal microscopy, magnetic circular dichroism, superparamagnetism, magnetic imaging, magnetite, SPION

## Abstract

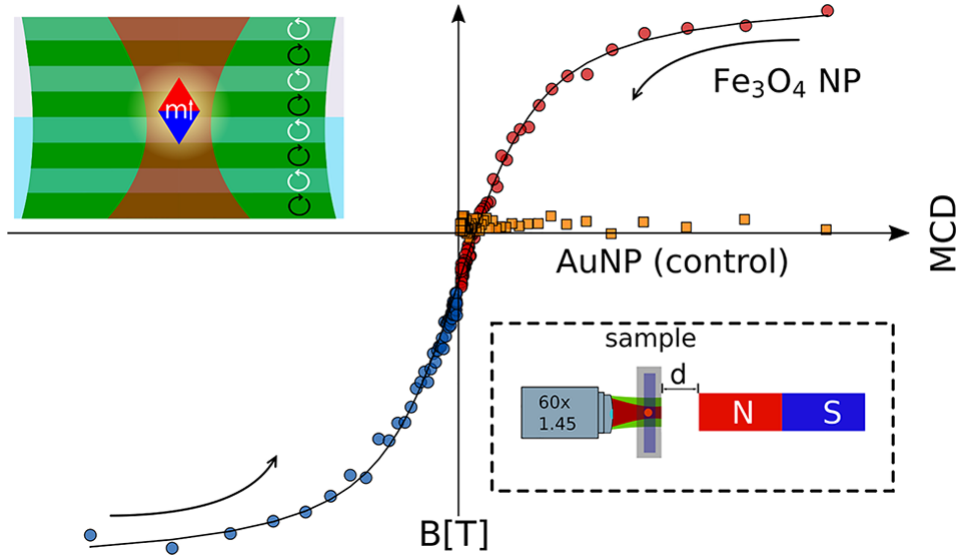

Magnetic imaging is a versatile tool in biological and condensed-matter physics. Existing magnetic imaging techniques either require demanding experimental conditions which restrict the range of their applications or lack the spatial resolution required for single-particle measurements. Here, we combine photothermal (PT) microscopy with magnetic circular dichroism (MCD) to develop a versatile magnetic imaging technique using visible light. Unlike most magnetic imaging techniques, photothermal magnetic circular dichroism (PT MCD) microscopy works particularly well for single nanoparticles immersed in liquids. As a proof of principle, we demonstrate magnetic CD imaging of superparamagnetic magnetite nanoparticulate clusters immersed in microscope immersion oil. The sensitivity of our method allowed us to probe the magnetization curve of single ∼400-nm-diameter magnetite nanoparticulate clusters.

## Introduction

Light–matter interaction in magnetic materials gives rise to unique magneto-optical phenomena such as the Faraday and Kerr effects. Ultrafast spectroscopy with femtosecond pulsed lasers enables the manipulation of magnetic properties via magnetization reversal^1^ and the demagnetization of ferromagnetic metallic thin films.^2^ Magnetic nanoparticles are promising candidates for applications in biomedicine, spintronics, and data storage.^3,4^ Magnetite (Fe_3_O_4_) nanoparticles, because of their size-dependent magnetic properties and biocompatibility, have been used for bioimaging and for photothermal^5^ and magnetothermal cancer treatment.^6^ For magnetic data storage, the minimum size of a magnetic bit is limited by the so-called “superparamagnetic limit”.^7,8^ Superparamagnetism occurs in ferromagnetic and ferrimagnetic materials. Bulk magnetite exhibits ferrimagnetic behavior. Single-domain magnetite nanoparticles of sufficiently small size randomly flip their magnetization directions, on the time scale of laboratory experiments (seconds to hours), when the magnetic energy barrier is on the order of or smaller than the thermal energy.^9^ When the measurement time is longer than the average time between two magnetization flips, the particle appears to carry no average magnetic moment. Like paramagnetic particles, superparamagnetic particles can be magnetized under an external magnetic field but typically exhibit much larger susceptibility. To study the magnetic phenomena of single nanoparticles, one can use nonoptical devices such as SQUIDs (superconducting quantum interference devices) and MFMs (magnetic force microscopes) or X-ray beam techniques such as XMCD (X-ray magnetic circular dichroism). Conventional SQUID magnetometers detect the net signal from large ensembles of nanoparticles, averaging out the size-dependent magnetic properties of the nanoparticles. For imaging purposes, scanning-SQUID microscopy can be used to image the magnetic flux from individual particles, in some cases with sub-100-nm resolution.^10^ Owing to their exceptional sensitivity, down to individual single-molecule magnets,^11^ SQUIDs are therefore widely used in the study of magnetic nanostructures;^12^ however, they require a cryogenic environment, complex probes, and electronics, which can be difficult to implement for many applications. MFM is a considerably simpler technique with excellent spatial resolution and can operate under ambient conditions. However, MFM also faces drawbacks such as topographic cross talk and the magnetic distortions caused by the strong stray fields of the probe.^13^ Furthermore, when studying samples in external magnetic fields, the sample and the probing tip are influenced by the field.^14^ XMCD provides high spatial resolution and sensitivity in the study of single nanoparticles,^15^ but it requires access to a beamline.

Magnetic circular dichroism (MCD) spectroscopy^16,17^ is an optical technique that exploits the polar Kerr effect.^18^ Until now, conventional visible-light MCD spectroscopy has been used only to investigate magnetic nanoparticles^16^ or (bio-) molecules^17^ in solutions containing an ensemble of many nanoparticles. The investigation of size- and shape-dependent magnetic properties, however, demands single-particle resolution. XMCD and electron holography measurements of single particles show that the magnetic properties depend not only on their size but also on their shape and on temperature.^15,19^ For instance, cobalt and iron particles in the range of 8 to 20 nm can exhibit both ferromagnetic behavior and superparamagnetic behavior at room temperature.^20^ Distinctions in magnetic behavior due to shape and size cannot be made by measuring an ensemble of nanoparticles, yet they are of vital importance to their applications in biomedical sciences and spintronic devices. Here, we overcome the limitation of visible-light MCD to ensemble measurements by implementing our newly developed photothermal circular dichroism (PT CD) microscopy^21,22^ for the magnetic imaging of single nanoparticulate clusters. To image the magnetization of nanoparticles, PT MCD is considerably simpler than XMCD or scanning SQUID, making it far more accessible. Our method directly measures the absorption of individual particles via their PT responses. Because the absorption linearly scales with the particle’s volume, we can access the particle’s size. The MCD signal, which we can determine separately via PT MCD measurements, displays a linear dependence on the particle’s magnetization.^18,23^ Our measurements therefore allow us to simultaneously access the size and the magnetization of individual particles.

**Figure 1:**
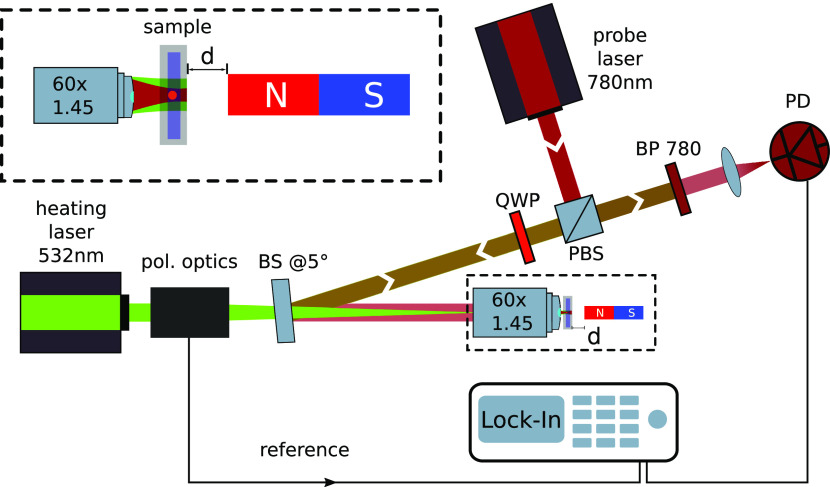
Schematic setup of the photothermal circular dichroism microscope. The 532 nm continuous wave (CW) heating laser beam is passed through a combination of polarization optics that modulates the polarization of the light between left and right circularly polarized light at ∼100 kHz. The 780 nm CW probe laser is passed through the combination of a polarizing beam splitter (PBS) and a quarter-wave plate (QWP) and combined with the heating beam at the 50/50 beam splitter (BS) at an angle of about 5°. The sample is illuminated with the heating beam in a Koehler configuration, whereas the probe beam is focused at the sample through an oil-immersion objective (NA = 1.45). The collected probe light is filtered from the heating light with a band-pass filter (BP 780). The photothermal signal is isolated by a lock-in amplifier. A long cylindrical permanent magnet is placed perpendicular to the sample plane at a variable distance *d* to apply a magnetic field to the sample. The inset shows an enlarged view of the heating and probe beam illumination and the position of the magnet relative to the sample. To flip the magnetic field direction, the magnet’s poles are flipped. A polarization optics unit consists of two polarization modulators driven at two different frequencies ω_1_ and ω_2_. An additional set of static birefringent plates and a polarizer enable polarization and amplitude modulation of the heating beam. Details are described in previous work.^22^ A reference signal at the sum frequency ω_1_ + ω_2_ of the two modulators is sent to the lock-in amplifier.

Figure 1 shows a scheme of our PT MCD setup. The setup is a photothermal microscope^24^ augmented with polarization modulation optics in the heating arm to facilitate photothermal circular dichroism measurements.^21^ We use a dual modulation of the polarization because it offers an excellent rejection of cross talk between linear and circular dichroism.^22^ By modulating either the intensity or the polarization of the heating beam, this setup can access absorption signals such as the total absorption (PT) and LD (linear dichroism) and CD. We use a Koehler configuration for the heating beam illumination, but we strongly focus the probe beam. Thereby, the optical resolution at the probing wavelength (780 nm) remains diffraction-limited, and the polarization state of the heating light (532 nm) remains well-preserved. This heating wavelength corresponds to intervalence charge transfer transitions of magnetite (Supporting Information). The technical details of the setup are explained in ref (22). In the circular dichroism mode, our technique measures the differential absorption between left and right circularly polarized light and therefore is most sensitive to magnetic moments that are parallel (or anti parallel) to the propagation direction of the light. To measure PT CD in the presence of an external magnetic field (PT MCD), a long cylindrical permanent magnet (NdFeB, 3 mm diameter, 10 cm length) is used to provide an out-of-plane magnetic field at the sample such that the light propagation is parallel to the magnetization direction (optical *z* axis). The magnetic field strength and sign can be varied by altering the distance and by flipping the orientation of the magnet relative to the sample.

## Results and Discussion

Here we study 400-nm-diameter magnetite nanoparticulate clusters (Chemicell GmbH) in a variable magnetic field. The tunable magnetic field enables us to discern MCD from geometric CD. Geometric CD is induced by the chiral structure of an object.^25^ In contrast to geometric CD, MCD in ferro- and ferrimagnetic materials results from the polar magneto-optic Kerr effect.^18,23^ The Kerr effect is induced by magnetic perturbations of the electronic states involved in optical transitions. It results from the interaction between electrons that provide magnetic moments and those that have large spin–orbit coupling.^18,26^

A single-crystalline magnetite particle of 400 nm diameter is expected to have multiple magnetic domains, and it will exhibit magnetic remanence. The particles used here, however, are clusters of sub-15-nm single crystals. At room temperature, magnetite particles of such size exhibit superparamagnetic behavior. When they form clusters in a wet chemical process, they can maintain their superparamagnetic behavior because of weak magnetic coupling between the individual subunits.^19,27,28^

**Figure 2:**
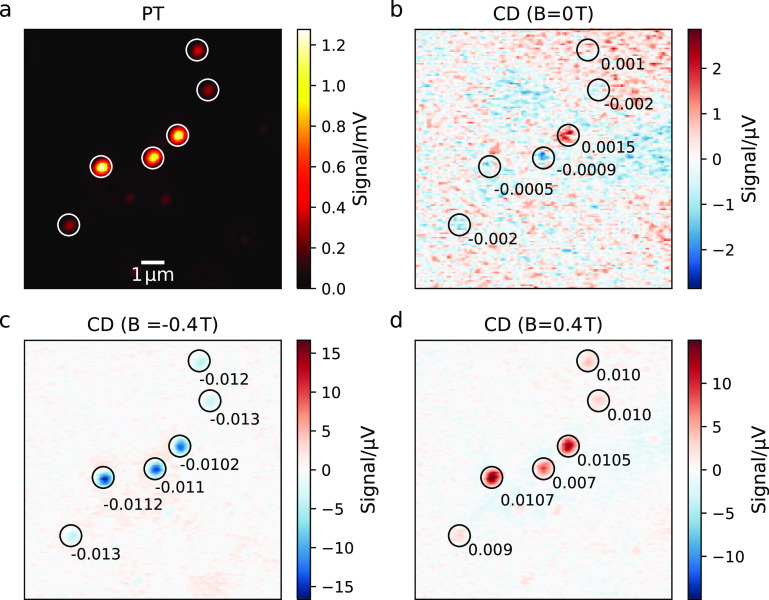
(a) Photothermal and (b–d) circular dichroism measurements of 400-nm-diameter magnetite particles exposed to different external magnetic fields. The actual size of the three brighter particles in the center is measured by correlative SEM imaging and falls into the range of 300–400 nm (Figures S1 and S2 in the SI). For sizing details, see Figures S1 and S2 in the SI. The integration time is 20 ms per pixel. The magnetic field strengths are (b) 0, (c) −0.4, and (d) 0.4 T. Individual particles exhibiting considerable MCD are marked with circles and numbers to indicate their *g* factors. At an external field of 0.4 T, the *g* factors are close to 1% for most particles.

To show that PT MCD can indeed provide images of single nanoparticles with magnetic contrast and to discern magnetic effects from shape effects (geometric chirality), we spin-coated the 400-nm-diameter magnetite particles on a glass surface at very low surface coverage (∼1 NP/10 μm^2^). We then obtained a series of four images as displayed on a selected example in Figure 2(a–d): (a) a photothermal image, which allows us to estimate the particles’ volume due to the linear relationship between the absorption cross section and volume; (b) a photothermal CD image in the absence of an external magnetic field, which allows us to determine the geometric CD of individual particles; and PT CD images obtained with axial magnetic fields of (c) positive and (d) negative sign. The axial magnetic field gives rise to a polar Kerr effect which can be detected by our setup via PT MCD.

**Figure 3:**
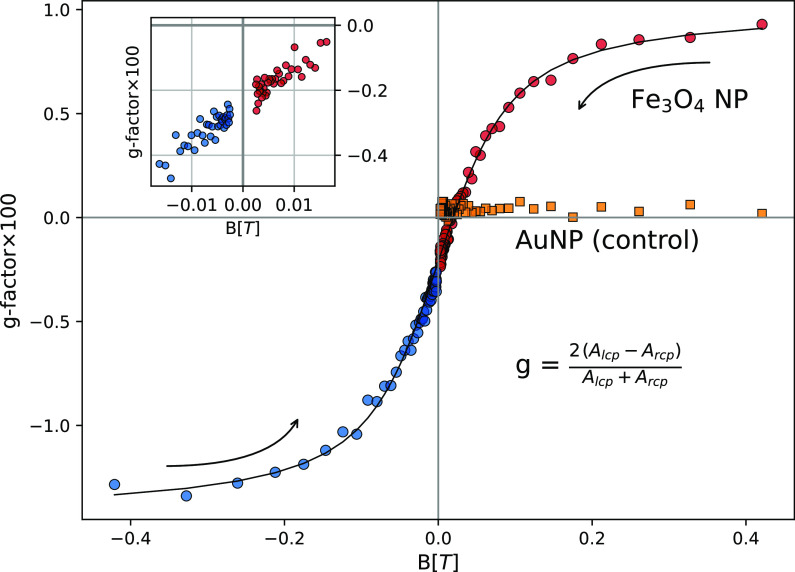
Magnetization curve of a single ∼400-nm-diameter magnetite particle measured by PT CD in hexadecane. The shape displays superparamagnetic behavior. The integration time is 1 s per point. The inset shows a magnified view of the magnetite NP’s magnetization curve at small fields. Arrows indicate the sweep direction of the magnetic field (strong to weak). The orange data points show a reference measurement on a 100-nm-diameter gold nanoparticle, here only for positive magnetic fields. The solid line is a fit with a Langevin function: (coth *x* – 1/*x*, where *x* = *μB*/*k*_B_*T*).^30^ The resulting magnetic moment of the composing nanoparticles is about 10 000 Bohr magnetons (μ_B_).

The PT scan (Figure 2a) indicates the broad size distribution of the particles. On the basis of the correlated SEM images (Figure S1 in the SI), we estimate the size of the three bright particles to be about 400 nm. Upon application of an external magnetic field, we find a strong contrast in the CD images (Figure 2c,d), indicating the presence of a net magnetic moment in the particles, which generates a strong MCD signal. We find that all particles exhibit the same CD sign and that, upon inversion of the magnetic field, we invert the sign of the CD signal, corroborating the MCD nature of the signal. We then compare the relative magnetic susceptibilities of the individual magnetite particles by calculating their *g* factors, defined as
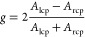
1where *A*_lcp_ and *A*_rcp_ are the absorptivities for left- and right-handed circularly polarized light, respectively. We can simply retrieve the *g* factor by calculating the ratio of CD over PT signals. The MCD signal is proportional to the magnetic moment, and the PT signal is proportional to the volume of the particle and thus to its number of unit cells. By taking their ratio, we obtain a value that scales with the magnetic moments per unit cell. We find that all particles have similar *g* factors, close to 1% in a magnetic field of 0.4 T, indicating a similar magnetic susceptibility. Figure 2(b) shows a CD scan in the absence of a magnetic field. We find that even in the absence of a magnetic field some particles exhibit CD. We assign this offset CD to geometric chirality that can occur in quasi-spherical particles^21,29^ and is different from particle to particle (Figures S5 and S6). Thanks to the excellent SNR of the measurements in Figure 2, we are not limited to measurements of the MCD close to the saturation magnetization of the magnetite particles. This motivated us to obtain the magnetization curve of a single ∼400 nm magnetite particle. We did this by focusing on a single 400-nm-diameter particle and measuring its MCD signal while the magnetic field’s strength and sign were varied by altering the magnet’s distance from the sample and flipping its orientation. To obtain the magnetic field strength as a function of distance, we performed a calibration measurement (SI, Figure S4) using a gaussmeter (Hirst Magnetic Instruments GM08). The resulting magnetization curve of a single magnetite particle is displayed in Figure 3.

The shape of the curve displays superparamagnetic behavior, as evident from the absence of remanent magnetization. The field we apply here (∼0.43 T) is larger than the saturation field of our nanoparticulate clusters (with a composing particle size of 8–13 nm)^31^ and below the saturation field of bulk magnetite.^32^ We fitted the magnetization curve with a Langevin function, *L*(*x*) = coth *x* – 1/*x*,^30^ where *x* = *μB*/*k*_B_*T*,μ is the magnetic moment, *k*_B_ is the Boltzmann constant, and *T* is the absolute temperature of ∼400 K). From this fit, we extracted an average magnetic moment of about 10 000 Bohr magnetons (μ_B_) for the nanoparticles composing the nanoparticulate cluster. On the basis of the nanoparticle size of 8–13 nm given by the manufacturer, the unit cell length of 0.84 nm, and the saturation magnetization of 32 μ_B_ per unit cell, we obtain a total magnetic moment of ∼10 000–40 000 μ_B_, which is in good agreement with our measurements. To exclude possible effects of the external magnetic field on the optical elements that may induce artificial CD, we performed a reference measurement on a 100-nm-diameter gold nanoparticle (orange data points in Figure 3). Gold is diamagnetic and therefore has a very small magnetic susceptibility.^33^ The small susceptibility should result in a negligible MCD response compared to that of magnetite. If the CD signal of the magnetic particles was due to the external magnetic field affecting the setup (i.e., the objective), we would also expect a response of the gold nanoparticles upon application of an external magnetic field. The shape of the gold nanoparticle’s PT CD curve shows that this particle had no significant response to the external magnetic field, within experimental noise. Together, these observations provide strong evidence that the observed strong MCD response of our magnetite particles is indeed induced by their magnetic moment.

The shape of the curve and the saturation magnetization that we find are in reasonable agreement with ensemble measurements for other magnetite nanoparticles;^34^ however, the strength of the MCD effect (*g* factor) that we observe is 1 order of magnitude larger than that found elsewhere in ensemble measurements.^35,36^ One reason for the difference in the *g* factor could be the different subunit sizes of the particles used in these studies, which were between 3.4 and 6.9 nm compared to 8–13 nm for our particles. Smaller nanocrystals exhibit a smaller saturation magnetization because of a magnetic dead layer.^31^ Another possible reason for the difference could be the different measurement modality. While references (35) and (36) contain measurements made in transmission geometry, thereby probing extinction, we employ PT-contrast probing absorption. Extinction measurements and absorption are not in general equivalent because the extinction measurement also entails a considerable scattering contribution.

## Conclusions and Outlook

We have demonstrated a PT-based optical imaging method that enables the study of single-particle magnetization via PT MCD. The excellent sensitivity of PT imaging allowed us to obtain single-particle magnetization curves. Our single-particle and absorption-based measurements revealed *g* factors that are 1 order of magnitude larger than the ones found by ensemble extinction-based studies.^35,36^ The images presented in Figure 2 were obtained by scanning the sample with a piezo-stage and thus require long image acquisition times. Recent advances in the field of wide-field PT imaging,^37−39^ which use cameras instead of confocal scanning, could open the possibility for faster image acquisition with magnetic contrast. PT imaging is particularly well suited for studying the absorption of small particles because the absorption scales with the volume. From our signal-to-noise ratios and the available laser powers of the probe and heating lasers, we estimate that the magnetic moments of single-domain magnetite particles with sizes down to 20–50 nm can be studied with our technique. We believe that photothermal magnetic circular dichroism (PT MCD) is a promising technique for future studies of magnetic nanoparticles because it is easy to implement in existing PT setups and does not suffer from the drawbacks of complex instrumentation and restrictive demands on experimental environments imposed by methods such as XMCD, MFM, and scanning SQUIDS.

## Supporting Information Available

Correlated SEM images of nanoparticulate clusters; PT of 20 nm magnetite particles; calibration of magnetic field vs distance; and magnetic-field-dependent PT-CD curves (PDF)
