# Supplementary material for: Imaging the Magnetization of Single Magnetite Nanoparticle Clusters via Photothermal Circular Dichroism
Source: Nano Lett. 2022 Apr 14;22(9):3645–50. doi: 10.1021/acs.nanolett.2c00178 (PMC9101077; doi:10.1021/acs.nanolett.2c00178)
Supplement: Supplementary file 1 — nl2c00178_si_001.pdf [file nl2c00178_si_001.pdf]

Supporting Information: Imaging the Magnetization of Single Magnetite Nanoparticle Clusters via Photothermal Circular Dichroism

Patrick Spaeth,[†,‡] Subhasis Adhikari,[†,‡] Kaveh Lahabi,[†] Martin Dieter Baaske,[†] Yonghui Wang,[†,¶] and Michel Orrit[*,†]

†*Huygens-Kamerlingh Onnes Laboratory, Leiden University, 2300 RA Leiden, Netherlands*

‡*These authors contributed equally*

¶*School of Mechatronics Engineering, Harbin Institute of Technology, Harbin, 150001, P. R. China*

E-mail: orrit@physics.leidenuniv.nl

Correlated SEM images of nanoparticulate clusters

Fig S1 shows a SEM image of the same area as the images of Fig 2 in the main text. The particles were specified to be 200 nm by the manufacturer (Chemicell GmbH). The sample surface is partially sullied with glass debris that probably came during the manufacturing process of markers on the glass slide (ibidi Gridded Glass Coverslips). The diameter of the encircled particles is significantly larger than 200 nm. However, the SEM scan, which was performed under 30° of the sample, also reveals that the particles are not spherical in shape. In fact they are rather flat. The three marked particles on the top right, for example, have

similar strength in PT signal and therefore should have the same volume. From the SEM images the particle looks smaller in diameter than the lower ones but appears significantly higher. From the tilted SEM images and the estimated height we estimate the volume of the particles to be equivalent to a sphere of ~400 nm in diameter.

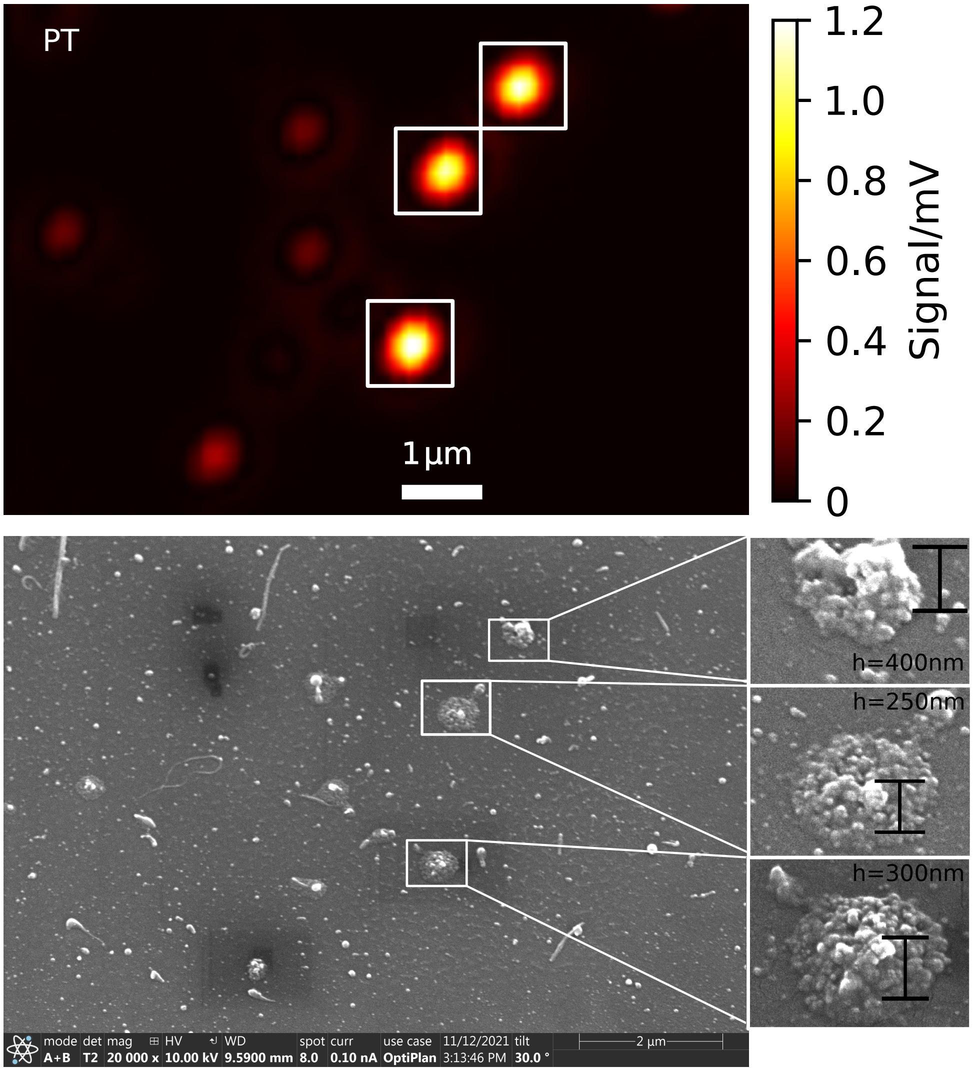

Figure S1: Correlated SEM images of the scanned area in Fig 2 of the main text. The top panel shows the PT image of the same sample region, which was rotated and mirrored compared to Fig 2 to fit with the SEM micrograph in the lower panel. The square boxes are guides for the eye. The particles' diameter is much larger than the 200 nm given by the manufacturer, however, the tilted SEM scan (30°) indicates that the particles are rather flat. The red bar indicates the estimated heights of the particles.

PT of 20 nm magnetite particles

Due to the small tilt of the SEM scans in Fig S1 (30°) and the crude shape of the particles we wanted to recheck the particle's size by comparing their absorption with well characterized 20 nm-diameter (Nanocomposix) magnetite particles. Based on the PT signal of the 20 nm-diameter magnetite particles, assuming that the absorption scales linearly with the volume, we can extrapolate the size of the supposedly 200 nm particles. The PT scans of three areas of a sample of spin-coated 20 nm-diameter magnetite particles are shown in Fig S2. Note the presence of a weak background PT signal that probably occurs due to weak absorption in the glass slide. This is only visible due to the weak absorption of the magnetite NP's and the relatively large heating power ($\sim$60 mW/µm^2). Fig S3 (c) shows a histogram of the PT signals of 193 magnetite NP's and (d) a TEM micrograph, provided by the manufacturer. The absorption scales linearly with the particle's volume. Accordingly the histogram of the cubic root of PT signals scales linearly with the particle size. Fig S3 (a) and (b) show the histogram of the cubic root of PT signals and the histogram of diameters measured by the manufacturer *via* TEM, respectively. The shape of the histogram of particle sizes based on the PT measurements agrees well with the histogram based on the manufacturer's TEM measurements (c,d). We can therefore assume that the center of the histogram in (a) corresponds to the center of the histogram in (b).

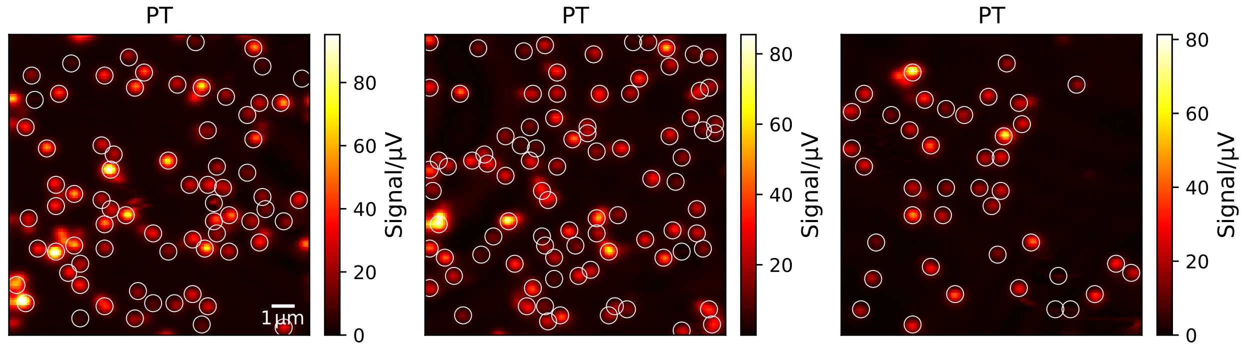

Figure S2: PT scans of 193 ∅20 nm particles from 3 different areas on the sample. The white circles are guides for the eye.

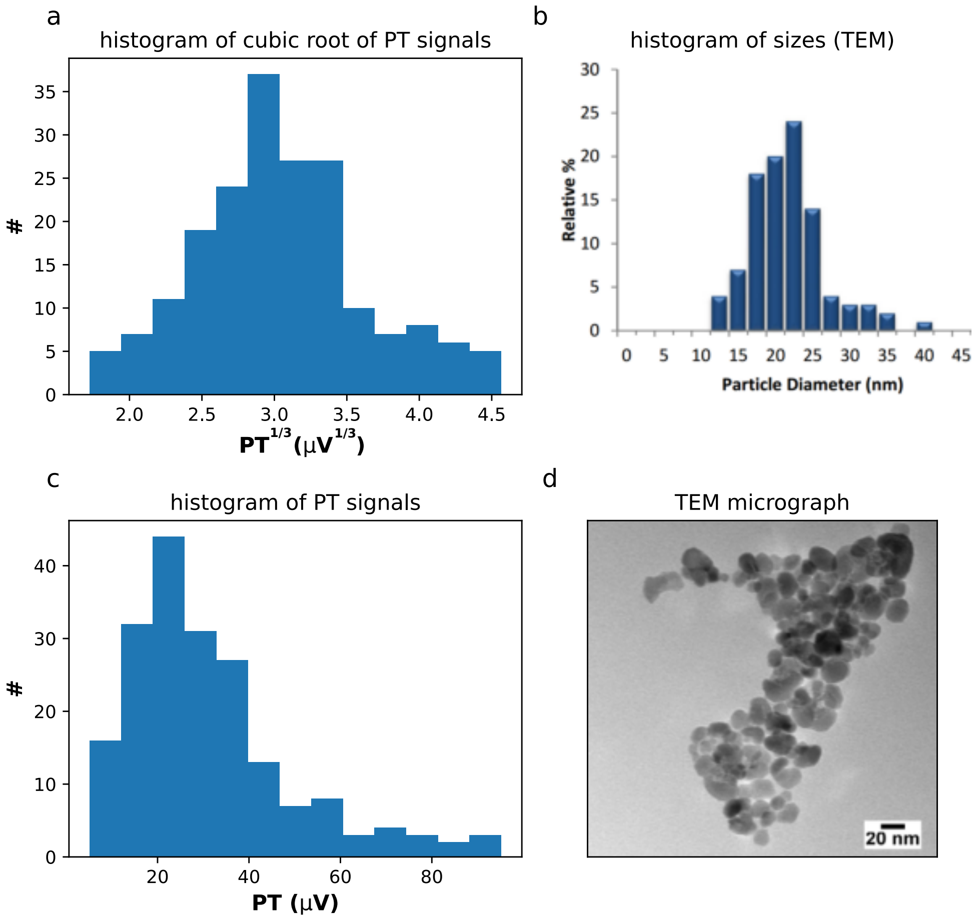

Figure S3: (a) and (c) histogram of PT signals of 193 ∅20 nm nanoparticles. b) size histogram from the manufacturer (NanoComposix) based on TEM imaging from the same particle batch. (d) TEM micrograph of the particles.

Based on the ratio of optical powers ($\times48$) and the ratio of the respective PT signals ($\times1/52$) we estimate the size of the supposedly 200 nm particles to be $\sim$300 nm. We therefore believe that the size of 400 nm that we found by SEM imaging is reasonable.

The optical absorption of magnetite in the visible is very intricate and difficult to interpret because of the complexity of the crystal (24 iron atoms per unit cell, three different types of iron ions), and because of the multiple atomic levels of transition metals. According to reference,[S1] transitions at the optical wavelength of 532 nm have a dominant inter-valence charge transfer (IVCT) character.

Calibration of magnetic field *vs* distance

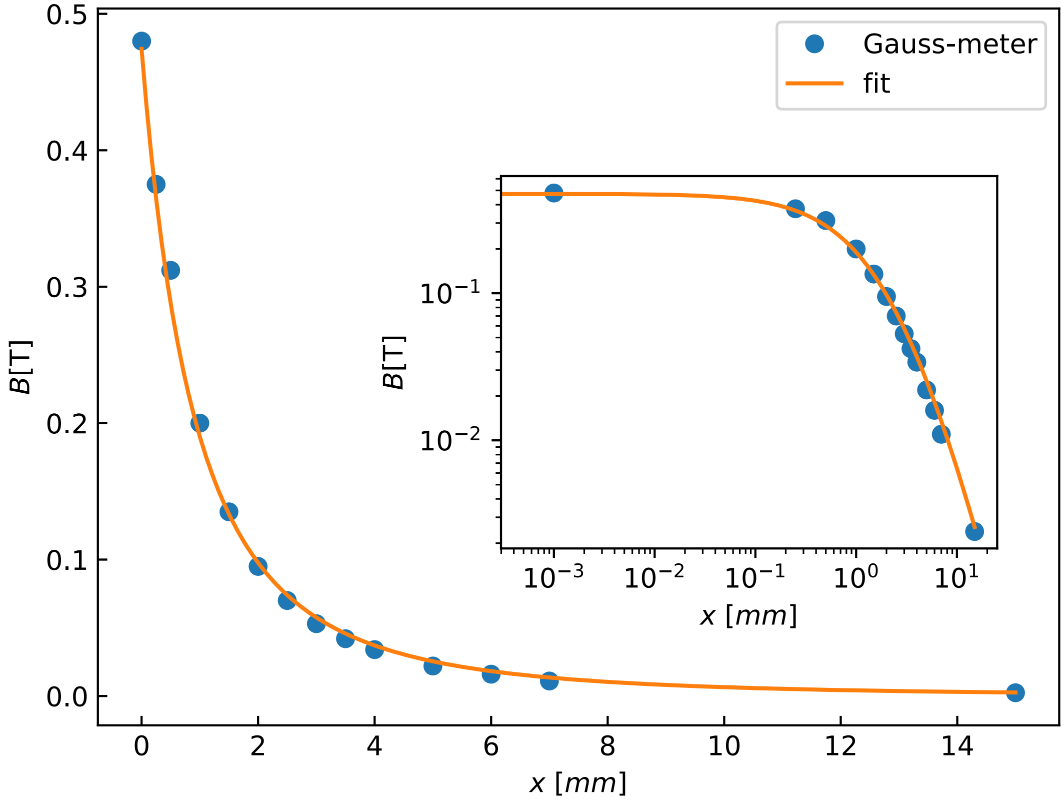

Figure S4: Measured dependence of magnetic field on distance (blue) and a phenomenological fit (orange). The magnetic field was measured wit a Gaussmeter. The insert shows a double logarithmic plot of the measured and fitted field dependence on distance.

Fig S4 shows the magnetic field dependence on distance of the permanent magnet we used to vary the magnetic field, measured with a Gaussmeter (Hirst Magnetic Instruments GM08) and the fitted data that is used to interpolate the field values at the positions relevant for the measurements of Fig 3 in the main text. We fit the data with the following phenomenological function:

$$B(x) = \frac{a}{(x+b)^2} \cdot \frac{1}{(1+x/c)} \tag{1}$$

For small distances we assume a $a/(x+b)^2$ dependence of the field, since the magnet is very long (monopole limit) with respect to its diameter. To improve the fit for larger distances where dipole effects come into play, we multiply by a factor of $1/(1+x/c)$. The respective values of a,b and c are: 1.85 $\mathrm{T{\cdot}mm^2}$,1.97 mm and 10.04 mm.

Magnetic-field-dependent PT-CD curves

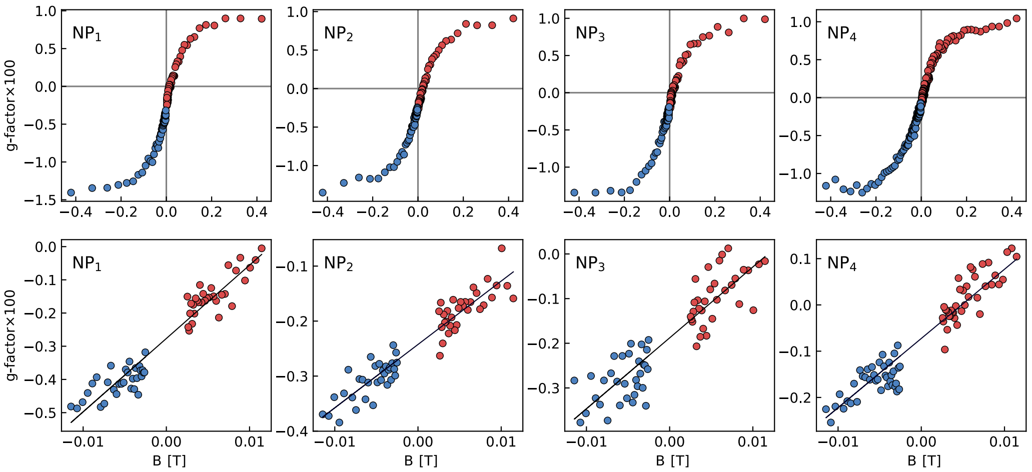

Figure S5: Magnetic-field-dependent circular dichroism curves of four different magnetite particles of ∼400 nm in diameter. The lower row shows a magnified view of the magnetization curve at smaller magnetic fields with a linear fit.

Fig S5 shows the magnetic field dependent PT-CD curves of four magnetite particles (NP1-NP4). All particles show similar g-factors indicating similar magnetic susceptibility. We can observe that the two parts of the magnetization curve (red and blue) seem to meet at zero magnetic field, within noise. This indicates the absence of a remanence magnetization. The offset values all have the same sign. This might give rise to the assumption of a bias in the CD measurements, due to the particles themselves or due to the method. We have shown in our recent work[S2] that our optical method is not subject to artificial bias signals. To rule out biasing effects of the particles we used the same sample as for the magnetization curve measurements, and performed a MCD scan including many particles.

Fig S6 a) shows a PT and three CD images with b) zero, c) positive and d) negative magnetic fields applied. When the magnetic field is turned off (Fig S6(b)) we still observe some residual CD signal that we relate to geometric chirality. We observe both positive and negative CD signals at zero external field. We therefore conclude that the four negative

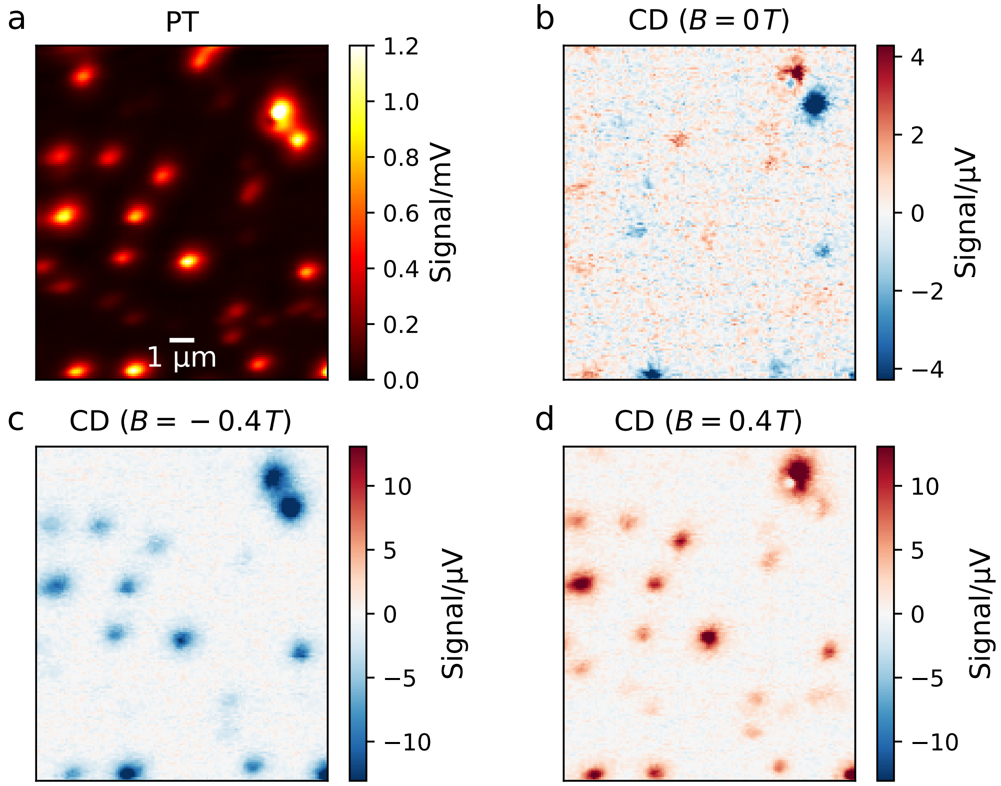

Figure S6: Photothermal (a) and photothermal CD images (b-d) of nominally 200 nm superparamagnetic magnetite particles at different external magnetic fields.

offset signals in Fig S5 are not systematic.

References

(S1) Yao, H.; Ishikawa, Y. Finite Size Effect on Magneto-Optical Responses of Chemically Modified Fe3O4 Nanoparticles Studied by MCD Spectroscopy. *The Journal of Physical Chemistry C* **2015**, *119*, 13224–13230.

(S2) Spaeth, P.; Adhikari, S.; Baaske, M. D.; Pud, S.; Ton, J.; Orrit, M. Photothermal Circular Dichroism of Single Nanoparticles Rejecting Linear Dichroism by Dual Modulation. *ACS Nano* **2021**, *15*, 16277–16285.
